# A Study of Vitamin D and Its Correlation With Severity and Complication of Congestive Heart Failure: A Systematic Review

**DOI:** 10.7759/cureus.28873

**Published:** 2022-09-06

**Authors:** Mohammad Hazique, Kokab Irfan Khan, Prasana Ramesh, Suthasenthuran Kanagalingam, FNU Zargham Ul Haq, Nishok Victory Srinivasan, Aujala Irfan Khan, Ghadi D Mashat, Safeera Khan

**Affiliations:** 1 Internal Medicine, California Institute of Behavioral Neurosciences & Psychology, Fairfield, USA; 2 Research, California Institute of Behavioral Neurosciences & Psychology, Fairfield, USA; 3 General Surgery, California Institute of Behavioral Neurosciences & Psychology, Fairfield, USA; 4 Pediatrics, California Institute of Behavioral Neurosciences & Psychology, Fairfield, USA

**Keywords:** inflammation, cardiac remodelling, renovascular hypertension, vitamin d supplementation, low level of vitamin d serum., myocardial infa, congestive heart faiulre

## Abstract

Many studies have shown that vitamin D is a crucial modulator of hypertension and cardiovascular illness, including heart failure. Heart failure (HF) is still the leading cause of mortality worldwide. Patients with heart failure who have low vitamin D levels experience worse outcomes, which associate with known clinical correlations and biomarkers. Additionally, patients with low vitamin D levels are more likely to have diabetes, hypertension, atherosclerosis, and other precursor conditions to heart failure. There are some hints in recent experimental research on how vitamin D can have cardioprotective effects. Vitamin D supplementation might improve ventricular remodeling in heart failure patients, however, this is still unclear. It aims to evaluate the association between vitamin D and congestive heart failure (CHF). This systematic review used research from the previous ten years (January 2012-2022) retrieved from the following databases: PubMed/PMC (PubMed Central)/Medline and Cochrane Library. Using the Preferred Reporting Item for Systematic Review and Meta-analysis (PRISMA) 2020 guidelines, removing duplicates, screening of title and abstract, application of eligibility criteria, and quality appraisal, 13 articles were retained for systemic review. There were 10 randomized controlled trials and three observational studies. Vitamin D supplementation lowers serum inflammatory marker levels and improves the quality of life in CHF patients. Vitamin D treatment inhibits ventricular remodeling and improves cardiac function in a patient with CHF.

## Introduction and background

Congestive heart failure (CHF) is a cardiac dysfunction syndrome characterized by a reduced left ventricular ejection fraction (LVEF) associated with water and sodium retention. Muscle weakness and early fatigue are the two primary symptoms of CHF patients. The prevalence of CHF is approximately 1% to 3% in western societies. Morbidity and mortality of CHF increase progressively with age [1-3]. Cardiovascular disease is the most prevalent chronic illness worldwide and is responsible for 30% of fatalities, placing a significant strain on healthcare systems [4]. Congestive heart failure is the major cardiovascular illness impacted by vitamin D insufficiency, accounting for most hospitalizations, healthcare spending, morbidity, and death, predominantly affecting middle-aged and elderly adults [5]. Heart failure affects over 6.5 million persons as of 2017 and is a factor in one of eight fatalities. To be more precise, modest lifestyle modifications like consuming foods high in vitamin D and dietary supplements can modify outcomes for these people and, in some circumstances, avoid life-threatening situations [6].

The etiology of CHF is not well understood. However, altered intracellular handling of ionized calcium (Ca^2+^) seems to play an essential role in the impaired contractility of the myocardium. In isolated myocytes from patients with terminal heart failure, markedly transient reduced systolic ionized calcium and diastolic ionized calcium levels were increased. In addition, myocyte cells slowed the rate of diastolic decay of ionized calcium compared with heart cells from healthy subjects [7].

Although angiotensin-converting-enzyme inhibitor (ACEI)/angiotensin receptor blockers (ARBs) and aldosterone receptor antagonist medicines can lower the frequency of adverse cardiac events and improve cardiac function [8], heart failure (HF) is still the leading cause of mortality worldwide. As a result, there is a significant need for additional therapeutic approaches and methodologies. In addition, cardiovascular disease may arise from a lack of or insufficient vitamin D [9]. 

Several studies have found a striking correlation between vitamin D deficiency and the development of CHF. Studies have revealed that patients with CHF typically lack vitamin D and have a bad prognosis; in addition, vitamin D therapy may lower the death rate of CHF patients [10-13]. 

**Figure 1:**
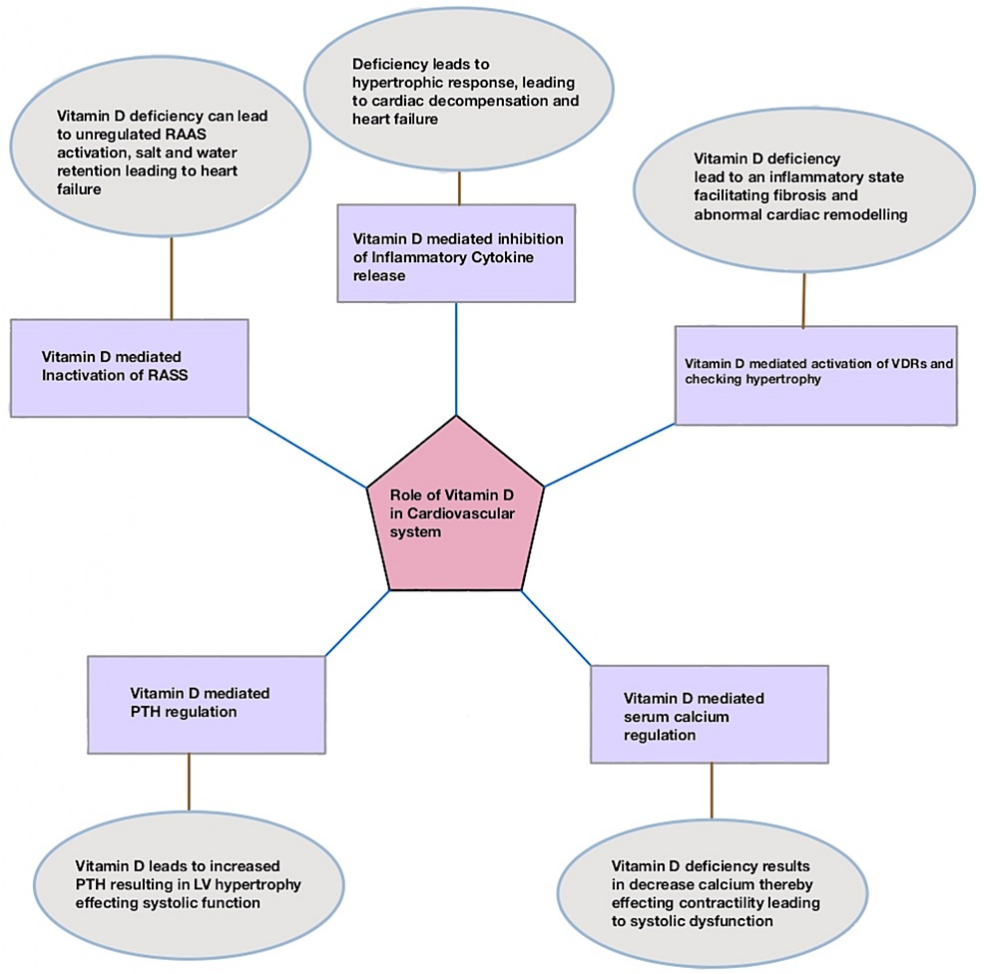
Role of Vitamin D in the Cardiovascular System Original illustration; RAAS: renin-angiotensin-aldosterone system; PTH: parathyroid hormone; LV: left ventricle; VDRs: Vitamin D receptors

Several randomized controlled trials (RCTs) have examined the benefits of vitamin D supplementation in CHF patients during the past several years. These studies examined clinical symptoms, cardiac function, cardiovascular events, and inflammation between vitamin D treatment and placebo (Figure 1). However, several of these experiments had conflicting findings and inconsistent conclusions. Only one meta-analysis of RCTs examined whether vitamin D supplementation is helpful for CHF patients; however, these studies included only adults, and their findings were inconsistent. To determine if vitamin D supplementation is advantageous for individuals with CHF and to assist in formulating these guidelines, we conducted a systematic review of the literature to summarize the available evidence regarding the possible cardiovascular harms and benefits of vitamin D.

## Review

Methods

Data Collection and Search Strategy

The Systematic review was conducted using the preferred Reporting Item for Systematic Review and Meta-Analysis - PRISMA 2020 [14]. From May 5, 2022, through May 30, 2022, a systematic search for papers and clinical articles published between 2012 and 2022 was undertaken on PubMed, PubMed Central (PMC), MEDLINE, and Cochrane. The standard search technique was used for papers indexed in PubMed, PMC, MEDLINE, and the Cochrane Library, and common keywords were used in the search approach. Table 1 summarizes the databases, search methodologies, and outcomes. 

**Table 1:** Data collection and Search Strategy Summarizes the databases, search methodologies, and outcome; PMC: PubMed Central

Databases	Search Strategy	Search Results
PubMed, PMC, MEDLINE	Congestive heart failure AND Vitamin D deficiency OR Vitamin D	362
Cochrane Library	Congestive heart failure AND Vitamin D deficiency OR Vitamin D	33

Study Selection Screening and Eligibility

**Figure 2:**
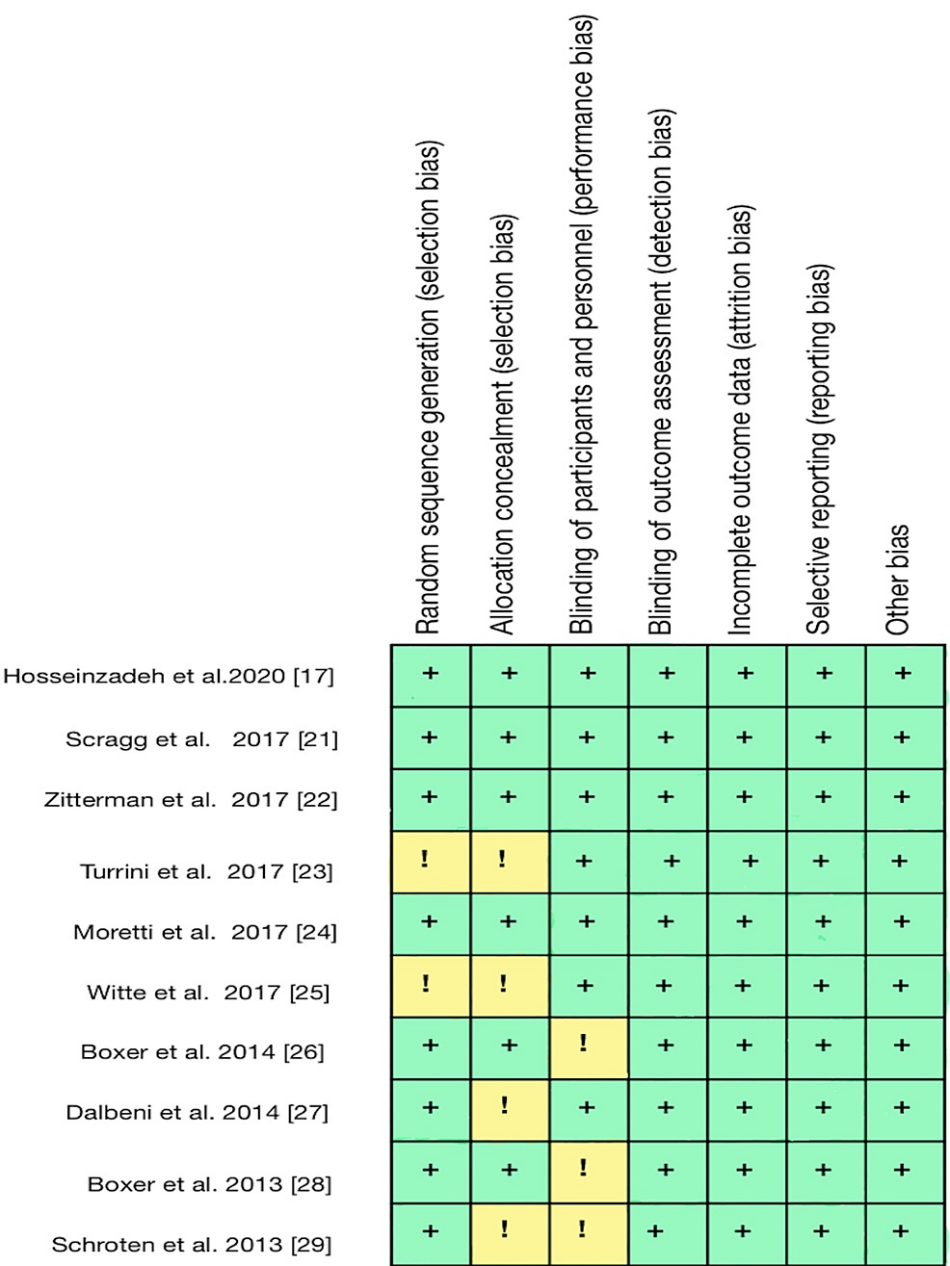
Cochrane assessment tool for Quality assessment of RCTs RCTs: Randomised control trials; Low Risk: (+); Unknown Risk: (!); [17-29]

EndNote (Clarivate 1.9, Philadelphia, United States) was used to filter and eliminate duplicates. The gathered publications were selected and separately checked for eligibility by title and then by abstract. After that, the inclusion and exclusion criteria were used to search for and evaluate the full texts of pertinent publications below (Table 2). Research studies (a) papers relevant to the question (b) published in the English language (c) randomized control trials (RCTs) assessing the effect of vitamin D in a patient with congestive heart failure (CHF) (d) full-text publications (e) focusing on humans (f) Age > 18 Years of age were considered. All research (a) irrelevant to the question, (b) not published in English, (c) unpublished literature (d) involving children were excluded. The number of studies selected for review after a quality assessment is summarised below (Table 2 and Figure 2).

**Table 2:** Quality Appraisal Tools employed for this study

Type of Study	Tool Used	Number of Studies
Randomized Clinical Trial (RCT)	Cochrane Bias Assessment Tool (figure 2) [15]	10
Cohort study	New Castle Ottawa scale [16]	3

Results

Study Search Outcome

**Figure 3:**
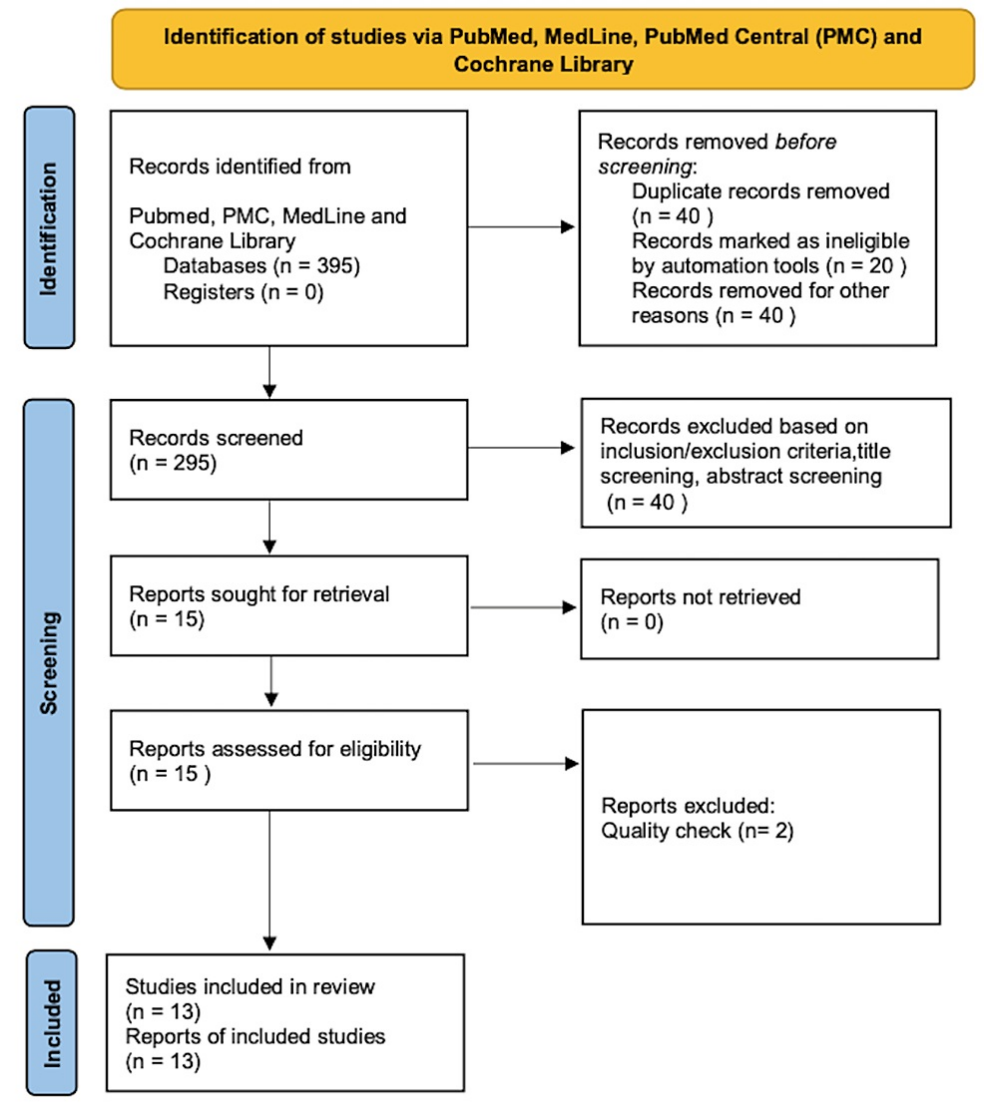
Preferred Reporting Item for Systemic Reviews and Meta-Analyses (PRISMA) 2020 flowchart PRISMA Flow Diagram Depicting the Screening Process for this review; PMC: PubMed Central; n: Number of records

After the initial screening, 395 published papers were gathered, and after reviewing the title, abstract, and full text and eliminating duplicate documents and studies that did not meet the inclusion criteria, 10 Randomised Control Trials with a total of 465 patients, 3022 cases in the vitamin D group and 3022 cases in the control group and three observational studies-were eventually included. The screening procedure is shown below (Figure 3). And the individual characteristics of the studies and outcomes included in the systemic review are presented in Table 3.

Study Characteristics

A total of 6,509 patients were identified from 13 published journal articles that underwent comprehensive selection analysis. The ultimate consensus on the effectiveness of vitamin D in congestive heart failure is according to the outcomes of each published journal below (Table 3).

**Table 3:** Summary of the individual characteristics of the studies and outcomes included in the systemic review RCT: Randomised control trial; BP: Blood pressure; 6MWT: six minute walk test; HF: Heart failure; CVD: Cardiovascular disease; MCS: Mechanical circulatory support; PTH: Parathyroid hormones; BNP: Brain natriuretic peptide; hsCRP: High-sensitivity C-reactive protein; LV: left ventricle; EF: Ejection fraction; PRA: Plasma renin activity.

Author and Year of Publication	Type of Study / Blinding	Vitamin D Dose	Intervention	Number of Patients	Follow up	Conclusion
Hosseinzadeh et al. 2020 [17]	Prospective RCT / Double-blinded	50,000 IU weekly	Vitamin D Placebo	21 18	8 Weeks	Compared to the placebo, there were no differences in BP or six-minute walk test (6MWT). In the intervention group, vitamin D levels considerably rose. Like other research, there has been no short-term improvement in the physical activity of HF patients.
Aparicio-Ugarriza et al. 2019 [18]	Retrospective Cohort study			284	4 Years	In Veterans with HF, a lack of vitamin D was independently linked to a greater likelihood of hospitalizations for all causes. Additionally, fragile HF patients had a considerably higher risk of all-cause hospitalizations than those who were not frail.
Cubbon et al. 2018 [19]	Prospective Cohort Study			1802	8 Years	Patients with chronic heart failure frequently experience vitamin D deficiency, which can last for years. In patients with chronic heart failure getting the highest quality of care and device therapy, a vitamin D deficiency is an independent predictor of increased mortality.
Costanzo et al. 2018 [20]	Prospective Cohort Study			24,325	5 Years	The patient with Vitamin D deficiencies had a higher chance of hospitalization.
Scragg et al. 2017 [21]	Prospective RCT / Double-blinded	200,000 IU load, followed 100,000 IU / monthly	Vitamin D Placebo	2558 2550	3.3 Years	Supplementing high doses of vitamin D every month will not prevent CVD. This finding does not support the monthly use of vitamin D supplements for this goal. Further research is needed to determine the effects of daily or weekly dosage.
Zittermann et al. 2017 [22]	Prospective RCT / Double-blinded	4,000 IU / day	Vitamin D Placebo	199 201	3 Years	A daily vitamin D intake of 4000 IU did not decrease mortality in individuals with severe heart failure but was linked to a larger requirement for MCS implants. Data suggest that relatively high vitamin D dosage for an extended period should be avoided
Turrini et al. 2017 [23]	Prospective RCT / Double-blinded	300,000 IU load, followed 50,000 IU/monthly	Vitamin D Placebo	17 16	6 Months	After three months, the therapy of vitamin D deficiency in HF patients increased the six-minute walk test (6MWT) and lowered PTH levels. However, treatment for vitamin D deficiency did not affect the results compared to the placebo arm.
Moretti et al. 2017 [24]	Prospective RCT / Double-blinded	10,000 IU /day	Vitamin D Placebo	17 19	8 Months	Vitamin D replenishment may enhance HF patients quality of life and aid in normalizing BNP, PTH, and hsCRP. More research is required to find out if dietary cofactors can improve vitamin D efficacy even further.
Witte et al. 2016 [25]	Prospective RCT / Double-blinded	4,000 IU / day	Vitamin D Placebo	80 83	1 Year	In patients receiving modern optimum medical treatment, a year of 100 mg of Vitamin D supplementation per day positively impacts LV shape and function. Still, it does not increase a six-minute walk TEST (6MWT). To discover whether these lead to better results, more research is required.
Boxer et al. 2014 [26]	Prospective RCT / Double-blinded	50,000 IU / week	Vitamin D Placebo	31 33	6 Months	In patients with HF and low blood vitamin D levels, vitamin D replenishment reduces aldosterone. Standard HF treatment may benefit from the addition of vitamin D. If vitamin D benefits HF patients in the long run, further research is needed to determine that.
Dalbeni et al. 2014 [27]	Prospective RCT / Double-blinded	4,000 IU / day	Vitamin D Placebo	18 18	6 Months	Six months of vitamin D therapy dramatically improves EF in elderly patients with HF and vitamin D insufficiency.
Boxer et al. 2013 [28]	Prospective RCT / Double-blinded	50,000 IU / week	Vitamin D Placebo	31 33	6 Months	Despite a significant rise in blood Vitamin D levels, exercise performance in HF patients was not improved by Vitamin D. Patients with HF should take vitamin D supplements following the same standards as healthy adults.
Schroten et al. 2013 [29]	Prospective RCT / Double-blinded	2,000 IU / day	Vitamin D Placebo	50 51	6 Weeks	Vitamin D deficiency and elevated PRA levels were common in CHF patients. Six weeks of 2,000 IU Vitamin D3 therapy improved serum vitamin D levels and lowered plasma renin and PRA levels.

Discussion

Role of Vitamin D in Congestive Heart Failure

Numerous researchers have sought to investigate the advantages of vitamin D administration in Congestive Heart Failure (CHF) patients. Still, their findings have only offered a few suggestions for improving the current standard of care. Several clinical trials employed a range of regimens (such as high dosage and low dose) for various lengths (long-term and short-term) and frequencies (daily, weekly, and monthly).

A three-year-long clinical study with 400 CHF patients receiving daily doses of 4000 IU of vitamin D was done with all-cause death as the primary outcome. The significance of advanced CHF was concluded by the fact that there was no difference in mortality between the treatment and control groups, with 19.6% of deaths in the treatment group and 17.9% in the placebo group. Additionally, their data showed no differences in hospitalizations, the requirement for cardiac resuscitation, or the necessity for transplantation. Further evidence that long-term vitamin D administration should be avoided was found in the intervention group's increased need for mechanical circulatory support implants [22]. This was the only randomized control trial (RCT) to show that reasonably high doses of 25-hydroxyvitamin D (25-OHD) were harmful to CHF patients. They attributed the negative consequences in these individuals to elevated plasma calcium concentrations.

On the other hand, a recent study examined the effects of short-term vitamin D treatment in heart failure patients [18]. They investigated the connection between physical activity and blood pressure (BP) and concluded that supplementation had no positive impact on BP or the six-minute walk test (6MWT) [17]. However, given the study's small sample size and the therapy's brief duration without maintenance, we should interpret these findings with care.

Another clinical trial was carried out to determine if monthly high-dose vitamin D supplementation, independent of blood 25-hydroxyvitamin D (25-OHD) status, can prevent cardiovascular disease in the general population [21]. A total of 5,108 individuals were chosen, and half received oral vitamin D_3_ at random, starting with a dose of 200,000 IU and then continuing with doses of 100,000 IU per month for a median of 3.3 years. Their main finding was that supplementation had no use and did not stop cardiovascular disease (CVD) events. They claimed that their monthly routine might cause this non-significance, while a daily or weekly regimen might be more efficient [21]. The significance of dosage frequency is therefore suggested.

Another RCT produced similar findings; in addition to a rise in ejection fraction, the research also revealed a favorable impact on systolic blood pressure [27]. But compared to Witte et al. study [25], this study's main flaw was its small sample size of 36 individuals with CHF and its use of supplements for six months.

Effect of Vitamin D on Renin Angiotensin Aldosterone System (RAAS), Serum 25-Hydroxyvitamin D, and Parathyroid Hormone in CHF

Regarding the impact of vitamin D supplementation on parathyroid hormone (PTH), one of the research studies found that six months of weekly high-dose treatment resulted in a significant rise in blood 25-OHD levels, with a proportionate drop in parathyroid hormone (PTH). However, this rise could not be clinically connected because there was no change compared to the placebo [26-28]. The limitations of this study were its small sample size of 30 patients and the short duration of six months. Another research that studies the impact of vitamin D on blood renin levels, the study by Schroten et al. [29], found that the treatment group's renin levels dropped dramatically. They concluded that because the absolute drop in renin was so tiny, they could not convert it into better outcomes for these individuals. However, given that persistently elevated plasma renin activity (PRA) is a characteristic that independently predicts poor outcomes in CHF patients, we might view decreased PRA as a beneficial interaction. Natriuretic peptides associated with the severity of CHF were not significantly affected [29]. This study's short six-week length could be a drawback. 

Effect of Vitamin D on the Prognosis of CHF Patients

According to the collected RCTs, vitamin D supplementation might enhance the quality of life (QOL) and lower the inflammatory response in CHF patients; however, it did not affect mortality or left ventricular function. And similar findings were evident in observational studies. A substantial group of researchers has linked poor clinical outcomes in CHF patients to vitamin D insufficiency. Most of these looked at hospitalization rates, mortality risk, effects on left ventricle ejection fraction (LVEF), and effects on physical activity. Recent research has identified vitamin D insufficiency as an independent risk factor for hospitalization in CHF patients. In addition, compared to veterans who were not frail, this risk was more prevalent in ailing veterans. The study's failure to demonstrate any correlation between mortality and deficit in its findings was a key finding [18]. The limited sample size and brief follow-up time may be the major causes of this lack of mortality impact.

A significant study from 2018 examined the relationship between vitamin D levels and the pattern of death rates and hospitalization risk in HF patients [20]. This study with vitamin D deficiency patients showed a considerable rise in cardiovascular hospitalizations, but no correlation with mortality, ejection fraction, or diastolic dysfunction was discovered [20]. Another long-term research looked at the causes of higher hospitalization and mortality rates in HF patients with vitamin D deficiency [19]. The study's data showed no correlation between the risk of hospitalization and death, but they did find that patients with defects died at considerably greater rates than participants without deficiencies [19]. Additionally, according to this study, a 2.72-fold rise in blood 25-OHD levels might reduce mortality by 14%.

Mechanism of Vitamin D for Heart Failure 

There is a relationship between vitamin D and heart failure (HF) prognosis. Due to excess ionized calcium (Ca^2+)^ in myocardial cells, HF affects the contraction and relaxation of the heart. On the other hand, vitamin D deficiency may affect the activities of Ca^2+^ in cardiac cells, leading to fibrosis, intra-organizational inflammation, and cardiomyocyte hypertrophy [24,29]. In addition, low vitamin D levels can cause inflammation, the renin-angiotensin system is activated, and endothelial dysfunction. Intriguingly, our subgroup analysis showed that patients lacking angiotensin-converting-enzyme-inhibitor (ACEI)/angiotensin receptor blockers (ARBs) had benefited more from vitamin D's protective effects on in-hospital mortality.

Furthermore, vitamin D's impact was more prominent in patients who did not use ACEI/ARBs, suggesting that their renin-angiotensin systems were activated [24,29]. This mechanism may imply the relationship between vitamin D and the cardiovascular system. Vitamin D deficiency may explain the relationship between vitamin D and prognosis in HF based on the fundamental understanding of these systems.

Limitations

Even though all trials included in this study were RCTs and few observational studies, there are still many limitations in this study. Future studies should examine if various vitamin D doses might be more advantageous, as well as different selection criteria. Future studies should concentrate on determining whether various vitamin D dosages would be superior, as well as defining multiple selection criteria (such as ejection fraction, vitamin D threshold, PTH threshold, etc.), and assessing additional echocardiographic parameters, the 6MWT, and cardiovascular mortality; According to the subgroup analysis, age stratification and if there is a lower ejection fraction may be sources of clinical heterogeneity in this study, which shows heterogeneity. Various recommended vitamin D doses have been reported in various trials, which may have an impact on research findings; hence, further studies are needed to investigate the link between vitamin D dosage and effect.

## Conclusions

Our systematic review assessed the function of vitamin D supplementation in Congestive Heart Failure (CHF) patients and clarified several adverse effects of low blood 25-hydroxycholecalciferol in these individuals. The current studies have shown that vitamin D has a weak and ambiguous impact on ventricular remodeling and cardiac function in patients with CHF. It cannot increase exercise tolerance or lower cardiac mortality. Daily vitamin D supplementation showed no discernible effect, and comparable outcomes were seen with high-dose monthly or weekly doses. Additionally, we discovered that inadequate vitamin D is linked to a higher risk of death, hospitalizations, and subpar clinical results. However, lingering lifestyle confounding variables could potentially account for this inverse connection. CHF patients with sufficient vitamin D have a decreased risk of all-cause death, even after considering potential confounding variables. These results support the need for a longer-term, fully recruited, randomized, placebo-controlled study of high-dose vitamin D3 supplementation in patients with CHF caused by left ventricular systolic dysfunction. Future studies would also need to include rigid clinical endpoints in line with our recent discovery that vitamin D supplementation results in beneficial cardiac remodeling. One cannot rule out the possibility of long-term advantages from vitamin D administration. We need clinical trials with more significant sample numbers and longer follow-up times to effectively establish benefits on mortality and hospitalization. Thus, further research is required on the ideal dose, frequency, optimum serum goal levels, and appropriate dose to enhance outcomes in CHF patients.
